# Pancreatic Neuroendocrine Tumor in the Setting of Dorsal Agenesis of the Pancreas

**DOI:** 10.1155/2016/3801962

**Published:** 2016-09-21

**Authors:** Samih Nassif, Cecilia Ponchiardi, Teviah Sachs

**Affiliations:** ^1^Boston University School of Medicine, 72 East Concord St., Boston, MA 02118, USA; ^2^Department of Pathology and Laboratory Medicine, Boston University School of Medicine, 72 East Concord St., Boston, MA 02118, USA; ^3^Boston University School of Medicine, Moakley Building, 3rd Floor, 830 Harrison Avenue, Boston, MA 02118, USA

## Abstract

Dorsal agenesis of the pancreas (DAP) is an uncommon embryological abnormality where there is absence of the distal pancreas. DAP is mostly asymptomatic, but common presenting symptoms include diabetes mellitus, abdominal pain, pancreatitis, enlarged pancreatic head, and, in a few cases, polysplenia. MRCP and ERCP are the gold standard imaging techniques to demonstrate the absence of the dorsal pancreatic duct. The literature on the association of pancreatic neoplasia and DAP is limited. We present the case of a pancreatic neuroendocrine tumor in a patient with dorsal agenesis of the pancreas, with a review of the related literature.

## 1. Introduction

Dorsal agenesis of the pancreas (DAP) is an uncommon embryological abnormality where there is absence of the distal pancreas. Here, we present the case of a 48-year-old female who was referred to our surgical oncology clinic for a pancreatic mass and was found to have concurrent DAP. We then discuss the embryology of DAP, as well as the most common clinical presentations of DAP and other established reports of DAP in the literature.

## 2. Case Report

A 48-year-old female was referred to the surgical oncology clinic for evaluation of a pancreatic mass. This was found incidentally on workup for an endometrial stromal sarcoma, for which she had undergone a total abdominal hysterectomy with bilateral salpingo-oophorectomy. The patient was asymptomatic.

**Figure 1:**
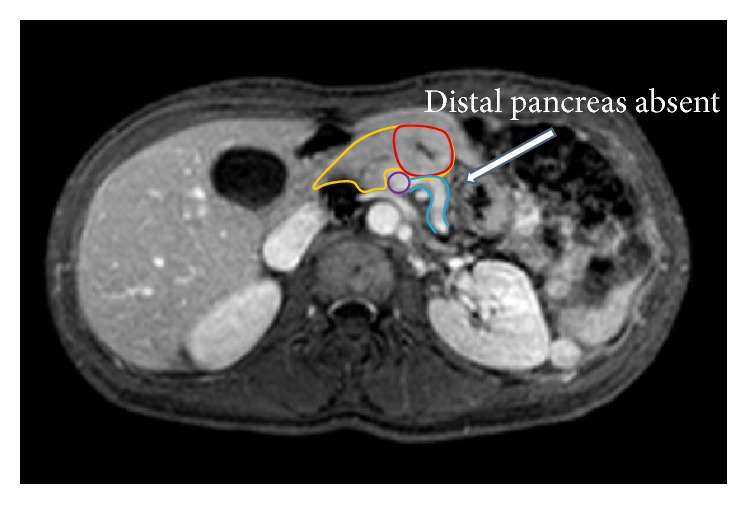
Axial image of an MRCP demonstrating the relevant anatomy of the tumor, vessels, and proximal pancreas, with the absence of the dorsal pancreas (white arrow). Outlines represent the pancreatic head and neck (yellow), the pancreatic neuroendocrine tumor (red), the superior mesenteric vein (purple), and the splenic vein (blue).

Her past medical history was significant for uterine sarcoma and for venous thromboembolism which led to a pulmonary embolus but was otherwise unremarkable. Her physical exam was unrevealing, as was her serum laboratory evaluation, with normoglycemia, normal hepatobiliary function, normal pancreatic enzymes, and no elevation in carbohydrate antigen 19-9, carbohydrate antigen 125, or carcinoembryonic antigen. CT and MRI imaging (Figure 1) revealed a mass at the neck of the pancreas, measuring 2.9 cm in its largest dimension, as well as the absence of the distal body and tail of the pancreas. The mass closely abutted the confluence of the portal vein and superior mesenteric vein, but there was no invasion. She underwent biopsy of this mass via endoscopic ultrasound which revealed features consistent with a well differentiated neuroendocrine tumor. The tumor was determined to be nonfunctioning given the absence of systemic symptoms and laboratory data to suggest hormone production.

**Figure 2:**
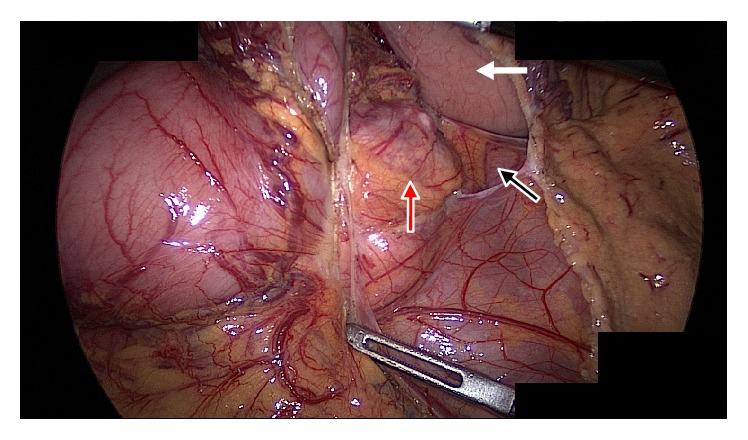
Laparoscopic image of the retroperitoneum as seen through a window created in the gastrocolic omentum. The stomach is elevated (white arrow), and the pancreatic neuroendocrine tumor (red arrow) can be seen with the absence of any pancreatic tissue distal to the tumor (black arrow).

**Figure 3:**
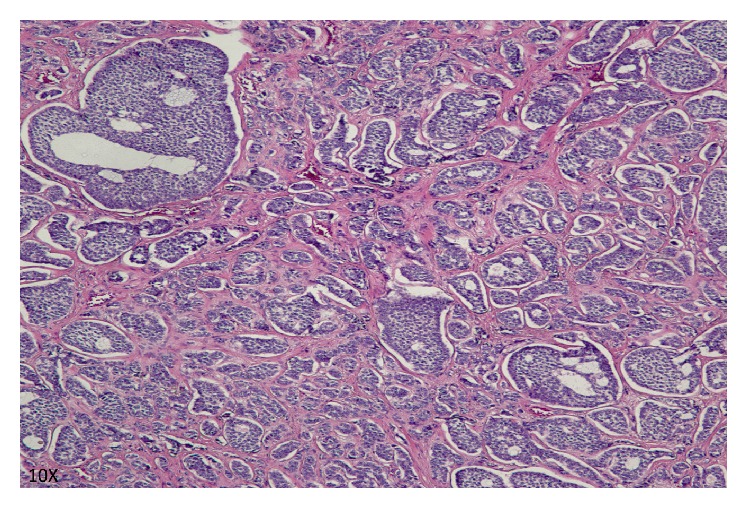
Histopathological description of the tumor. It is composed of multiple nests with hyalinized fibrovascular stroma. Tumor cells are relatively uniform with finely granular eosinophilic cytoplasm and centrally located round to oval nucleus with “salt and pepper” chromatin pattern. There were less than 2 mitoses per high-powered field. The tumor was chromogranin positive after immunohistochemical analysis (not shown).

The patient underwent resection of this mass via spleen preserving laparoscopic approach. Intraoperative images confirmed the absence of the distal body and tail of the pancreas (Figure 2). Negative margins were achieved with this resection, and the pancreatic head and uncinate process were preserved, as were the splenic vein and artery. The pancreatic parenchyma was transected using a linear cutting stapler, with a closed staple height of 1.5 mm, and the remnant pancreatic neck was buttressed with an omental patch. A 19 Fr fluted Blake drain was placed at the resection margin at the time of surgery. Final pathology revealed a grade 1 well differentiated pancreatic neuroendocrine tumor (Figure 3). Despite our intraoperative efforts to avoid it, the patient's postoperative course was significant for a pancreatic duct leak, which was well controlled by her drain, and she was discharged home on POD 4. Her drain was removed on POD 23. She had no evidence of diabetes or pancreatic insufficiency on follow-up evaluation. Her case was discussed at our multidisciplinary tumor board and no further treatment for this tumor was recommended.

## 3. Discussion

### 3.1. Embryology of the Pancreas

During the fourth week of gestation, the dorsal (cranial) and ventral (caudal) buds of the pancreas develop from the endoderm at the junction of the foregut and the midgut. While the dorsal bud develops only into pancreatic tissue (anterior head, body, and tail), the ventral bud also contributed to the liver, gallbladder, bile ducts, and ventral pancreas (posterior neck and head) [1]. The ventral pancreatic duct (duct of Wirsung) and the common bile duct thus share a common entry point to the duodenum at the major papilla. Eventually, the ventral bud rotates clockwise and fuses with the dorsal bud at the seventh week of gestation. At this time, the dorsal pancreatic duct (duct of Santorini) fuses with the ventral pancreatic duct to create the main pancreatic duct [2]. Islets of Langerhans primarily develop in the dorsal pancreas, at week twelve of gestation.

### 3.2. Dorsal Agenesis

Dorsal agenesis occurs when there is abnormal development of the dorsal pancreatic bud, but there is intact development of the ventral pancreatic bud. Thus, there is absence of the anterior head, body, and the tail of the pancreas with intact formation of the liver, gallbladder, bile ducts, and posterior neck and head of the pancreas. The dorsal pancreatic duct never forms, and pancreatic secretions course from the ventral pancreatic duct into the common bile duct and eventually pass through the major papilla into the second portion of the duodenum.

The first case of dorsal agenesis of the pancreas (DAP) was reported in 1911 as an autopsy finding, and since then there have been relatively few reported cases in the literature [1]. DAP can be complete or partial. In patients with complete DAP, the dorsal duct system and the body and the tail of the pancreas are all missing. However, in partial DAP, the accessory papilla, the terminal end of the main dorsal duct of Santorini, or the pancreatic body is present. Familial DAP has been described in association with other congenital deformities, as well as alone. The molecular basis for DAP is not well defined; however, certain homeobox genes have been associated with DAP in rodent models [10].

DAP can be asymptomatic due to exocrine and endocrine functional reserve in the remaining pancreas. However, given that most of the islets of Langerhans develop in the body and tail of the pancreas, diabetes mellitus can occur [1]. Other common findings in association with DAP include abdominal pain, pancreatitis, enlarged pancreatic head, and, in a few cases, polysplenia [3, 11]. Diagnosis of DAP requires demonstration of the absence of dorsal pancreatic tissue. CT can be useful as an initial study to delineate the size of the pancreas. MRCP and ERCP are the gold standard imaging techniques to demonstrate the absence of the dorsal pancreatic duct [12]. Treatment of patients with DAP is guided by the symptomatology with which they presented [1].

### 3.3. Pancreatic Neoplasia and Dorsal Agenesis

The association of pancreatic neoplasia and DAP has not been studied extensively; a PubMed search identified 10 such cases published since 2000 (Table 1) [2–9]. The mechanism of this association is uncertain however. Some theorize that DAP increases the risk of chronic pancreatitis, which in and of itself is a risk factor for pancreatic tumors.

Treatment of pancreatic neoplasia in the setting of DAP does not deviate from current management guidelines [13]. Surgical resection of pancreatic tumors in patients with DAP often requires resection of the remaining pancreatic tissue, with a high rate of insulin dependent diabetes mellitus and exocrine insufficiency. In our case, we were able to preserve the majority of the proximal pancreas, mitigating the risks of postoperative diabetes.

## 4. Conclusion

We present the case of a pancreatic neuroendocrine tumor in a patient with dorsal agenesis of the pancreas, which, to our knowledge, has not previously been reported in the literature. The patient presented with an asymptomatic, incidentally discovered pancreatic mass at the neck of the pancreas that was resected with negative margins via spleen preserving, laparoscopic approach. This is one of the few cases of pancreatic neoplasia identified in patients with dorsal agenesis of the pancreas (DAP), a rare developmental anomaly where there is absence of the distal pancreas. DAP is most commonly asymptomatic but can present with symptoms of new-onset diabetes mellitus, abdominal pain, or chronic pancreatitis. Because of its silent presentation, there are very few cases of DAP reported in the literature and even fewer cases of DAP with concurrent pancreatic neoplasia.

## Figures and Tables

**Table 1:** Cases of pancreatic neoplasia in patients with dorsal agenesis of the pancreas.

Reference	Presenting symptoms	Imaging modality to confirm DAP	Operation	Final tumor histology
Matsusue et al. 1984 [3]	Abdominal pain, weight loss, hyperglycemia	ERCP	Total pancreatectomy	Ductal adenocarcinoma
Nakamura et al. 2001 [4]	None	ERCP	Subtotal pancreatectomy	Solid pseudopapillary tumor
Ulusan et al. 2005 [5]	Abdominal pain, type II diabetes mellitus	CT abdomen and pelvis	Pancreaticoduodenectomy	Solid pseudopapillary tumor
Ulusan et al. 2006 [6]	Abdominal pain, jaundice, hyperglycemia	Unknown	Hepaticojejunostomy	Ductal adenocarcinoma
Rittenhouse et al. 2011 [2]	Abdominal pain, type II diabetes mellitus	ERCP	Pancreaticoduodenectomy	Ductal adenocarcinoma
Rittenhouse et al. 2011 [2]	Abdominal pain, weight loss, type II diabetes mellitus	CT abdomen and pelvis	Pancreaticoduodenectomy	Ductal adenocarcinoma
Rittenhouse et al. 2011 [2]	Elevated liver function tests, asymptomatic	ERCP	Pancreaticoduodenectomy	Ductal adenocarcinoma
Kapoor and Singh 2011 [7]	Painless jaundice, weight loss, cholangitis	Intraoperative pancreatogram	Pancreaticoduodenectomy	Ampullary carcinoma
Sannappa et al. 2014 [8]	Jaundice, weight loss	MRI abdomen	Total pancreatectomy	Periampullary adenocarcinoma
Mistry et al. 2015 [9]	Painless jaundice, type II diabetes mellitus	CT abdomen and pelvis	Pancreaticoduodenectomy	Ampullary carcinoma
